# Review of evidence implicating the plasminogen activator system in blood-brain barrier dysfunction associated with Alzheimer’s disease

**DOI:** 10.20517/and.2022.05

**Published:** 2022-01-29

**Authors:** Mei-Yun Tang, Fredric A. Gorin, Pamela J. Lein

**Affiliations:** 1 Department of Molecular Biosciences, School of Veterinary Medicine, University of California, Davis, CA 95616, USA.; 2 Department of Neurology, School of Medicine, University of California, Davis, CA 95616, USA.

**Keywords:** Bradykinin, matrix metalloproteinase, neuroinflammation, neurovascular unit, plasmin, tissue-type plasminogen activator

## Abstract

Elucidating the pathogenic mechanisms of Alzheimer’s disease (AD) to identify therapeutic targets has been the focus of many decades of research. While deposition of extracellular amyloid-beta plaques and intraneuronal neurofibrillary tangles of hyperphosphorylated tau have historically been the two characteristic hallmarks of AD pathology, therapeutic strategies targeting these proteinopathies have not been successful in the clinics. Neuroinflammation has been gaining more attention as a therapeutic target because increasing evidence implicates neuroinflammation as a key factor in the early onset of AD disease progression. The peripheral immune response has emerged as an important contributor to the chronic neuroinflammation associated with AD pathophysiology. In this context, the plasminogen activator system (PAS), also referred to as the vasculature’s fibrinolytic system, is emerging as a potential factor in AD pathogenesis. Evolving evidence suggests that the PAS plays a role in linking chronic peripheral inflammatory conditions to neuroinflammation in the brain. While the PAS is better known for its peripheral functions, components of the PAS are expressed in the brain and have been demonstrated to alter neuroinflammation and blood-brain barrier (BBB) permeation. Here, we review plasmin-dependent and -independent mechanisms by which the PAS modulates the BBB in AD pathogenesis and discuss therapeutic implications of these observations.

## INTRODUCTION

Alzheimer’s disease (AD) is recognized as the most common cause of dementia in the elderly, and over 6 million Americans are currently living with this disorder. In the United States, AD is the sixth leading single cause of death and the second most common contributing cause of death. The hallmark neuropathologic characteristic of AD is abnormal extracellular protein accumulation in the brain, notably the extracellular deposition of amyloid-β (Aβ) peptide generated from the improper cleavage of amyloid precursor protein (APP) that gives rise to Aβ monomers that aggregate into oligomeric Aβ fibrils and plaques, and intraneuronal neurofibrillary tangles (NF) comprised largely of hyperphosphorylated tau. These proteinopathies are associated with the loss of synapses and subsequent neuronal cell loss in the entorhinal cortex, hippocampus, and frontal cortex^[1–3]^, and currently, the biomarkers most commonly used in human AD studies are beta-amyloid 42, tau, and phospho-tau proteins in the cerebrospinal fluid. More recently, blood p-tau181 has been reported as being a useful biomarker for distinguishing AD from other dementias^[4]^. Thus, it has been widely posited that Aβ plaques and/or abnormal hyperphosphorylated tau protein accumulation are causally linked to the behavioral and neurologic symptoms of AD. However, therapeutic strategies for decreasing Aβ plaque load^[5,6]^, reducing Aβ production with BACE-1 inhibitors^[7]^, or inhibiting hyperphosphorylated tau aggregation^[8]^, have been largely unsuccessful in clinical trials over the past several years^[3]^. These failed clinical trials coupled with observations of age-related increases in Aβ deposition in cognitively intact individuals as well as evidence that Aβ plaque load does not closely correspond with cognitive decline in AD patients^[1,9]^ and neurofibrillary tangles are associated with severe cognitive impairment characteristic of late stages of AD^[10,11]^, have prompted research into alternative pathogenic mechanisms of AD.

It is now recognized that the extracellular deposition of Aβ and hyperphosphorylated tau triggers proinflammatory responses in microglia and astrocytes^[12–14]^. The neuroinflammatory response in AD has been described in detail in several recent reviews^[14,15]^, and it appears that neuroinflammation plays an important role in the early progression of AD^[16,17]^. Multiple investigators have shown that Aβ monofibrils, oligomers, and plaques activate gene expression of pro-inflammatory mediators in microglia and astrocytes^[13,16,18,19]^. While microglial phagocytosis of amyloid may be neuroprotective in the early stages of AD by promoting Aβ clearance^[20,21]^, microglial activation in later stages may promote the progression of AD^[1,16]^. Network-based integrative analysis of whole-genome gene-expression profiling and genotypic data obtained from late-onset AD and non-demented control brains identified the immune/microglia module as the molecular system most strongly associated with the pathophysiology of AD, and in particular, late-onset AD^[22]^. Microglial activation is thought to promote AD progression by (1) complement-mediated phagocytosis of synaptic structures to promote synapse loss; and/or (2) release of nitric oxide (NO) and proinflammatory cytokines, including TNF-α, IL-6, and IL-1β, that act as soluble synaptotoxic factors and induce “A1” neurotoxic astrocytes^[23–26]^. In support of these proposed mechanisms, microglial activation has been linked to increased synaptic loss and neurodegeneration in AD^[2,24,27]^, and pharmacologic inhibition of microglial proliferation in the APP/PS1 mouse effectively shifted microglia to an anti-inflammatory phenotype that was associated with decreased synaptic degeneration and improved memory^[28]^. In Alzheimer mouse models, early synaptic loss is associated with C1q complement tightly bound to AB plaques surrounded by neuronal atrophy from microglial phagocytosis^[29]^. Mononuclear phagocytes enter the central nervous system (CNS) signaled by chemokines (CXCL1), while the innate immune system also appears to contribute to the neuroinflammatory response to activated microglia in AD models^[30]^.

While the initial focus on the role of the immune response in AD pathogenesis has been on the brain’s intrinsic neuroinflammatory response, attention is now being directed to multiple systemic inflammatory disorders that accelerate or in some instances may be the primary trigger for neuroinflammatory responses that initiate and/or promote AD and other dementias^[31–34]^. Some of the observations that have stimulated this shift in focus include reports that young children chronically exposed to high levels of air pollution were found to have neuropathological hallmarks of AD upon incidental autopsy^[35,36]^, and evidence that type 2 diabetes/ metabolic syndrome and inflammatory bowel disease are associated with increased risk of developing AD^[15,37,38]^. The causal factors linking peripheral inflammatory conditions to AD are likely multifactorial and have not yet been clearly delineated; however, several mechanisms are emerging. Peripheral inflammatory conditions have been shown to (1) generate inflammatory cytokines that facilitate access of peripheral inflammatory lymphocytes into the CNS, most notably TNFα, IL-1β, and IL-6; (2) cause dysfunction of the blood-brain barrier (BBB); and (3) activate the plasminogen activator system (PAS), which has direct effects on the CNS and further facilitates BBB dysfunction. The remainder of this review will investigate the role of the PAS in mediating inflammatory crosstalk between the periphery and the brain and its potential role in AD pathogenesis.

## PLASMINOGEN ACTIVATOR SYSTEM

**Figure 1.**
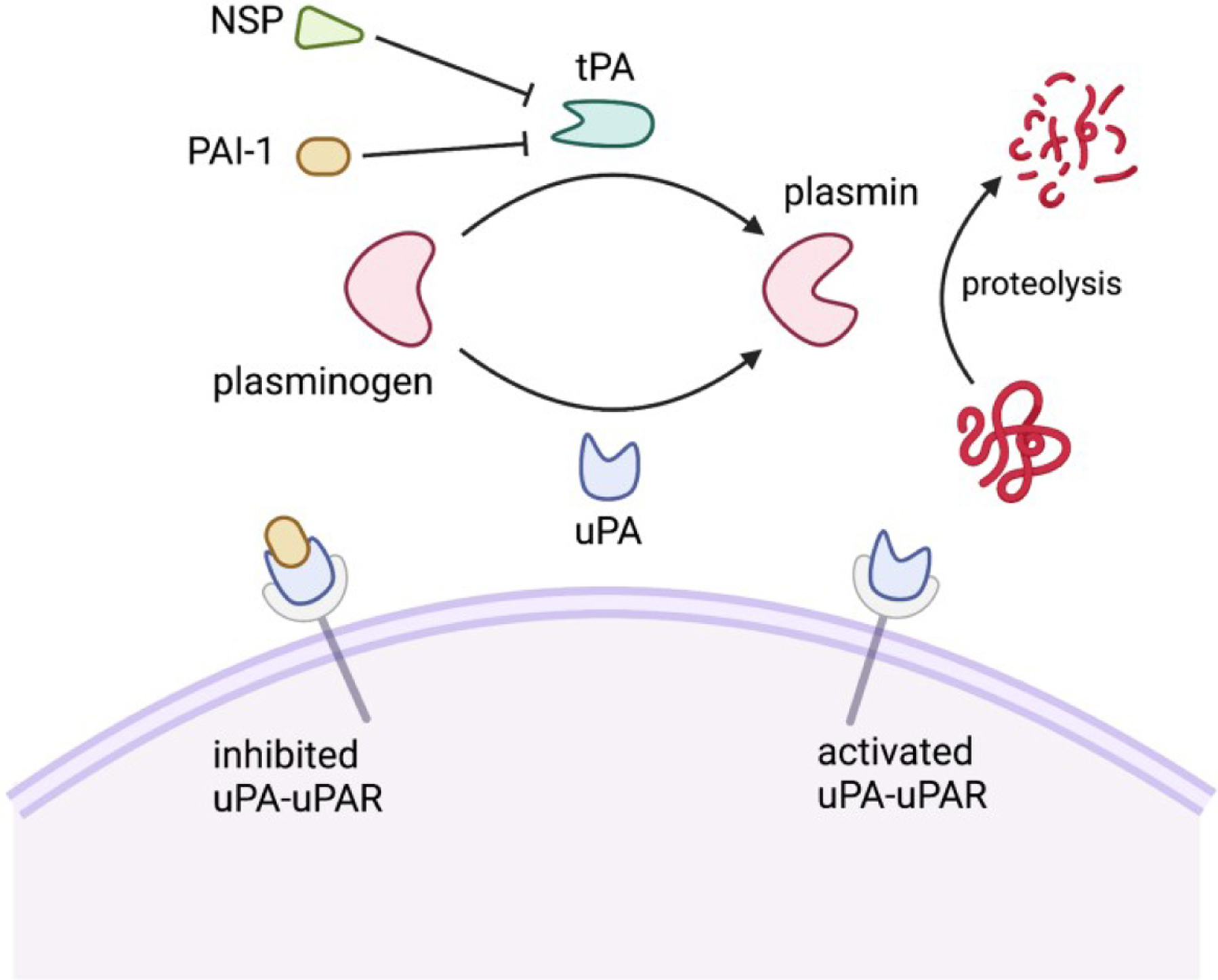
Schematic diagram of the molecular mechanisms of the plasminogen activator system. PAI-1: Plasminogen activator inhibitor-1; NSP: neuroserpin; uPA: urokinase-type plasminogen activator; tPA: tissue-type plasminogen activator; PLG: plasminogen; PLM: plasmin. Created with BioRender.com.

The plasminogen activator system (PAS) is comprised of a group of serine proteases, inhibitors, and binding proteins that control the activity of the serine protease plasmin [Figure 1]^[39]^. Plasmin plays a key role in the fibrinolysis cascade, catalyzing the final degradation of fibrin and various extracellular matrix proteins^[40,41]^. The zymogen plasminogen (PlG) is converted to activated plasmin by plasmin activators, which include tissue-type plasminogen activator (tPA) and urokinase-type plasminogen activator (uPA). tPA is primarily involved in intravascular fibrinolysis, activating plasminogen that is bound to polymerized fibrin. In contrast, uPA is secreted as a pro-enzyme whose active form is primarily localized on cell surfaces where it binds to the uPA receptor (uPAR). Plasminogen conversion by tPA and uPA in both the periphery and the CNS is tightly regulated by serine protease inhibitors (serpins). Serpins represent a superfamily of proteins with similar structures. Most relevant to this discussion are plasminogen activator inhibitor type 1 (PAI-1) and neuroserpin (NSP). PAI-1 irreversibly inhibits uPA or tPA by undergoing a large conformational change upon binding uPA or tPA that disrupts the active site of the plasmin activator and of PAI-1. In contrast, NSP preferentially inhibits tPA by forming an unstable complex that can release active tPA^[42]^. Reflecting the need for stringent regulation of the plasminogen cascade, free forms of activated plasmin activators, PAI-1, and NSP exist at very low concentrations with half lives in the order of minutes^[43,44]^.

### PAS in the periphery

The peripheral PAS plays a central role in mediating fibrinolysis, extracellular migration, cell signaling, cellular migration, and tumor growth, which has been reviewed in detail elsewhere^[45,46]^. The PAS converts inactive plasminogen to plasmin, a trypsin-like serine protease, via the catalytic activity of PA^[41]^. Plasminogen is primarily present in platelets in the plasma and liver. However, in mice, plasminogen mRNA has been found in the adrenal, kidney, brain, testis, heart, lung, uterus, spleen, thymus, and gut^[40,47]^. In the periphery, PAI-1 serves as the main suppressor of plasma fibrinolytic activity^[40]^. In the bloodstream, PAI-1 exists on its own in an active form, or as part of a complex with tPA or vitronectin, a glycoprotein that can convert PAI-1 into its active form. Elevated levels of PAI-1 are associated with metabolic syndrome and associated with increased risk of atherothrombosis and stroke^[48,49]^.

### PAS in the CNS

In the CNS, plasminogen is expressed at low levels by neurons in the hippocampus, cortex, cerebellum, as well as neuroendocrine tissues, but it is primarily transported to the brain via systemic circulation^[12,50,51]^. Plasminogen has been localized to the extracellular space, while the plasmin activators, tPA and uPA, have been localized to not only the extracellular space, but also to neurons and astrocytes. Both plasmin activators have been shown to modulate synaptic function when released into the synaptic cleft^[52–54]^. Membrane depolarization induces the rapid release of tPA from cerebral cortical neurons, which modulates neuronal plasticity, learning, stress-induced anxiety, and visual cortex plasticity^[55]^. tPA and uPA activities have been localized to well-defined areas of the brain^[56–59]^ and shown to participate in intracellular signaling that is independent of plasminogen activation (see below). tPA is the principal plasmin activator in the CNS with PAI-1 regulating its activity primarily in the extracellular space. NSP is primarily localized in neurons in the developing brain with very low levels detected in the mature CNS^[60]^, where it preferentially binds to and inhibits tPA^[61]^. Interestingly, mutations of NSP are associated with rare familial dementia characterized by neuronal inclusion bodies that are biochemically comprised of polymers of NSP^[62]^.

Plasmin activity has been shown to be upregulated in axonal growth and synaptic pruning, suggesting a role in brain development and regeneration that is not yet well understood^[50]^. While both tPA and uPA can mediate plasminogen activation in the CNS, plasminogen activation is primarily controlled by the tight regulation between tPA and PAI-1^[51]^. uPA has a low baseline expression in specific neurons and astrocytes in the normal brain, but is upregulated in pathologically inflammatory environments, such as multiple sclerosis and epilepsy^[50,51]^. Endothelial cells of microvessels in the brain contribute to the production of tPA, but tPA can also be expressed by glial cells, neurons, and infiltrating leukocytes, implicating a broad spectrum of tPA involvement in the brain. While tPA in the mature brain is detected primarily in neurons, its enzymatic activity is primarily restricted to the hippocampus, amygdala and hypothalamus^[63,64]^. The discrepancy between the expression of tPA mRNA and its areas of enzymatic activity is consistent with its trafficking and transport to mossy fiber tracts^[63,64]^.

The plasmin activators, tPA and uPA have been shown to play an important role in CNS function and dysfunction with some of their functions being independent of plasminogen^[65,66]^. Extracellular tPA participates in cerebellar motor learning^[67]^, remodeling in various nonneural tissues^[67]^, and neuronal regeneration following ischemic injury^[68]^. tPA also participates in the regulation of BBB permeability^[69,70]^. Neuronal uPA is present in lower levels than tPA, participating in neurogenesis in the developing brain^[71]^. Its release in the mature central nervous system triggers astrocytic activation^[53]^ and, like tPA, uPA promotes axonal and synaptic recovery following different forms of injury^[72]^. Both tPA and uPA are found in pre-synaptic vesicles that are released by calcium-dependent mechanisms^[52,54,55]^.

### The PAS is altered in AD

There has been longstanding interest in the role of the PAS in AD beginning with early reports that active plasmin efficiently digests Aβ peptides^[73–77]^ both *in vitro* and in rodent AD models^[19,73,74,76–81]^. In the AD brain, tPA is highly expressed in regions of AD plaques, and in AD models where tPA is genetically inactivated, there is an increased accumulation of Aβ, synaptic dysfunction and memory deficits^[78]^. However, the enzymatic ability of brain tPA and uPA to activate plasmin *in vivo* is thought to be prevented by irreversible binding to high levels of extracellular PAI-1 secreted by immune-activated microglia and astrocytes^[18]^. PAI-1 is minimally expressed in the normal brain or cerebral vasculature, but does increase with senescence^[82–84]^. Brain levels of PAI-1 are also markedly increased in APP/PS1 mice^[66]^ and the serum levels of PAI-1 are positively correlated with cognitive impairment in AD patients^[85]^. Consistent with the hypothesis that PAI-1 promotes AD pathology, genetic knockdown or small molecule inhibitors of PAI-1 reduced plaque formation in AD rodent models, and the small molecule PAI-1 inhibitor, PAZ-417, was shown to significantly improve hippocampal LTP and cognitive function in AD mice^[73,74,86,87]^. This finding was confirmed recently in an APP/PS1 AD mouse model using another small molecule PAI-1 inhibitor^[86]^.

Whether tPA primarily plays a beneficial or detrimental role in AD progression is debated. Several studies have demonstrated that tPA activation of plasmin enzymatically reduces Aβ accumulation^[78]^. Conversely, tPA has been shown to mediate excitotoxic neurodegeneration by activating plasmin and causing subsequent laminin degradation^[66,78]^. Independent of plasmin activation, tPA causes GSK3 activation, tau hyperphosphorylation, microtubule destabilization, and neurotoxicity in rodent hippocampal neurons^[88]^. It has also been shown to mediate amyloid-induced microglial activation^[89]^. Based on such observations, it has been proposed that tPA contributes to neurotoxicity, microglial activation, and tau hyperphosphorylation as part of a feed-forward inflammatory pathway^[73,88,89]^.

PAI-1 expression has been reported to be increased in the plasma^[85,90,91]^ and brain tissues of AD patients^[76]^. PAI-1 expression is not detected in normal healthy human brains but is sporadically present in aged brains^[84,92]^, and possibly linked to cerebrovascular disease. PAI-1 is the primary regulator of tPA in the CNS extracellular space and is a proinflammatory biomarker. Cytokines upregulate PAI-1 expression in microglia and astrocytes in human and animal models of AD^[18,93]^. The PAI-1 promoter is activated by TNF-α via an NFκB 5′ upstream element and directly activated by TGF-β1 via SMAD2/3 promoter binding sites^[82,94,95]^. When PAI-1 is complexed with low density lipoprotein receptor-related protein-1 (LRP-1), it signals changes in microglial morphology and motility that are consistent with microglial activation^[96–98]^. In patients with AD, plasminogen activator activity is reduced while PAI-1 and NSP are upregulated^[99]^. However, there are contradictory findings regarding measurements of PAI-1 and tPA in the CSF and serum of patients with AD^[76,92,100]^.

Congophilic amyloid angiopathy (CAA) is a vascular complication of AD in which Aβ40 plaques accumulate within the brain endothelium of cerebral arteries, arterioles and capillaries^[101]^. CAA can result in intracranial hemorrhages, cognitive impairment, or subacute inflammatory encephalopathy. tPA activation of endothelial NMDA receptors has been shown to regulate neurovascular coupling via nitric oxide-mediated regulation of cerebral blood flow. Elevated levels of brain PAI-1 impairs this tPA-dependent neurovascular coupling in Tg2576 AD mice, and pharmacologic inhibition of PAI-1 was shown to improve cognition in this animal model by selectively restoring neurovascular function while not affecting cortical amyloid plaques^[102]^.

### PAS modulates BBB integrity in AD

**Figure 2.**
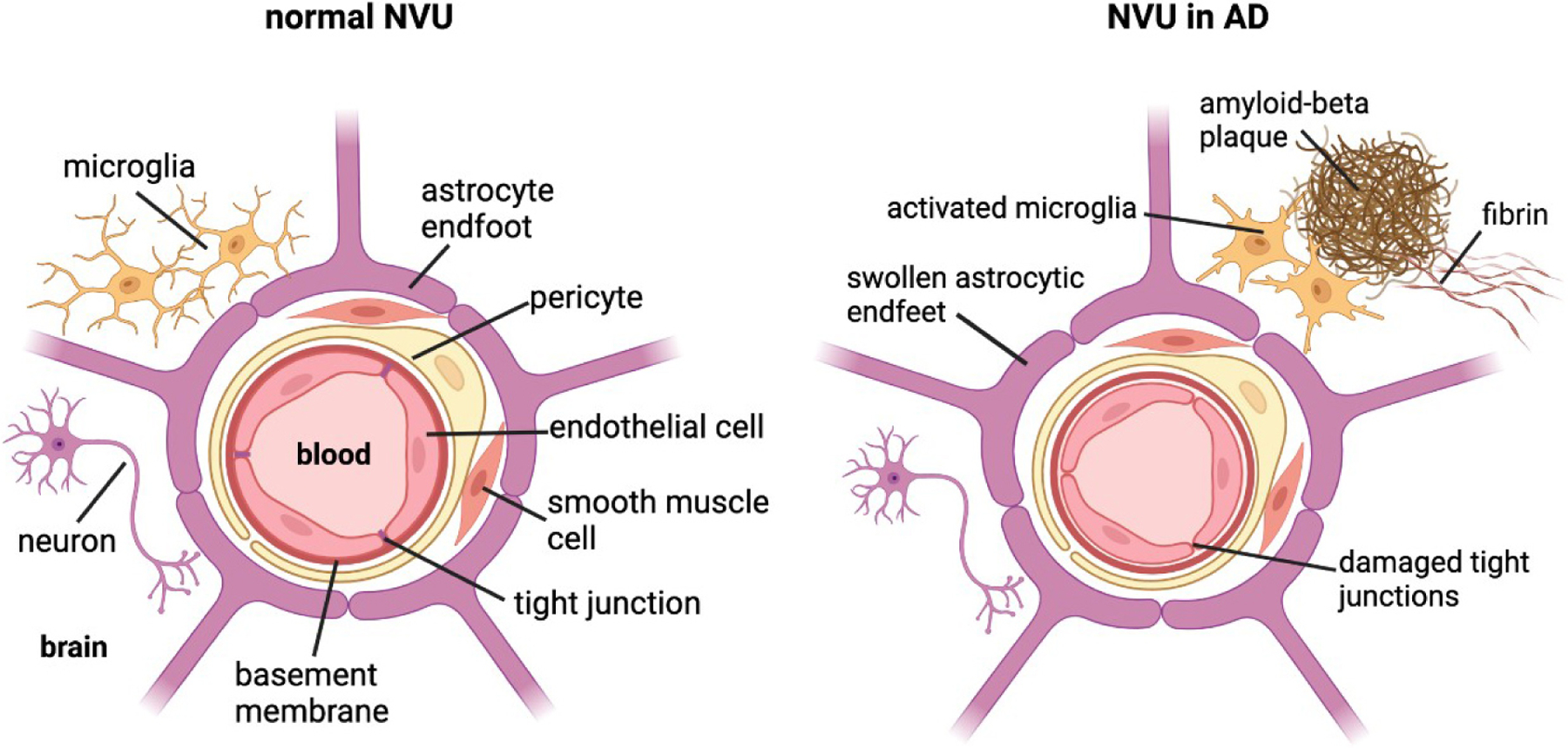
Cross-section of the neurovascular unit (NVU) in a normal brain *vs*. an Alzheimer’s disease (AD) brain. The blood-brain barrier (BBB) consists of endothelial cells joined by tight junctions, basement membrane, mural cells (i.e., pericytes and vascular smooth muscle cells), enclosed by astrocytic endfeet. Neurons and microglia closely associate with the BBB. In the AD brain, the NVU undergoes morphological and structural changes due to AD pathology. Amyloid-beta plaques complexed to fibrin result in neuroinflammation and BBB disruption, including activated microglia, swollen astrocytic endfeet, and compromised tight junctions. Created with BioRender.com.

There is increasing evidence identifying BBB leakage as an early sign of cognitive dysfunction, as well as evidence linking BBB dysfunction to AD pathogenesis^[103,104]^ and its neuroinflammatory pathology^[33,105]^. However, the mechanisms underlying BBB dysfunction in AD are currently not well-elucidated. The BBB is part of the neurovascular unit (NVU) in the brain, which consists of endothelial cells (ECs), mural cells, including vascular smooth muscle cells and pericytes, basement membrane, glia cells including astrocytes and microglia, and neurons [Figure 2]. The ECs of the BBB are a distinct characteristic of the NVU due to their tight junctions and lack of fenestrae. This allows the ECs to regulate the selective transport and metabolism of substances from blood to brain and vice versa, thereby separating the microenvironment of the brain parenchyma from changes in circulating ion and metabolite concentrations in the systemic circulation^[105]^.

**Figure 3.**
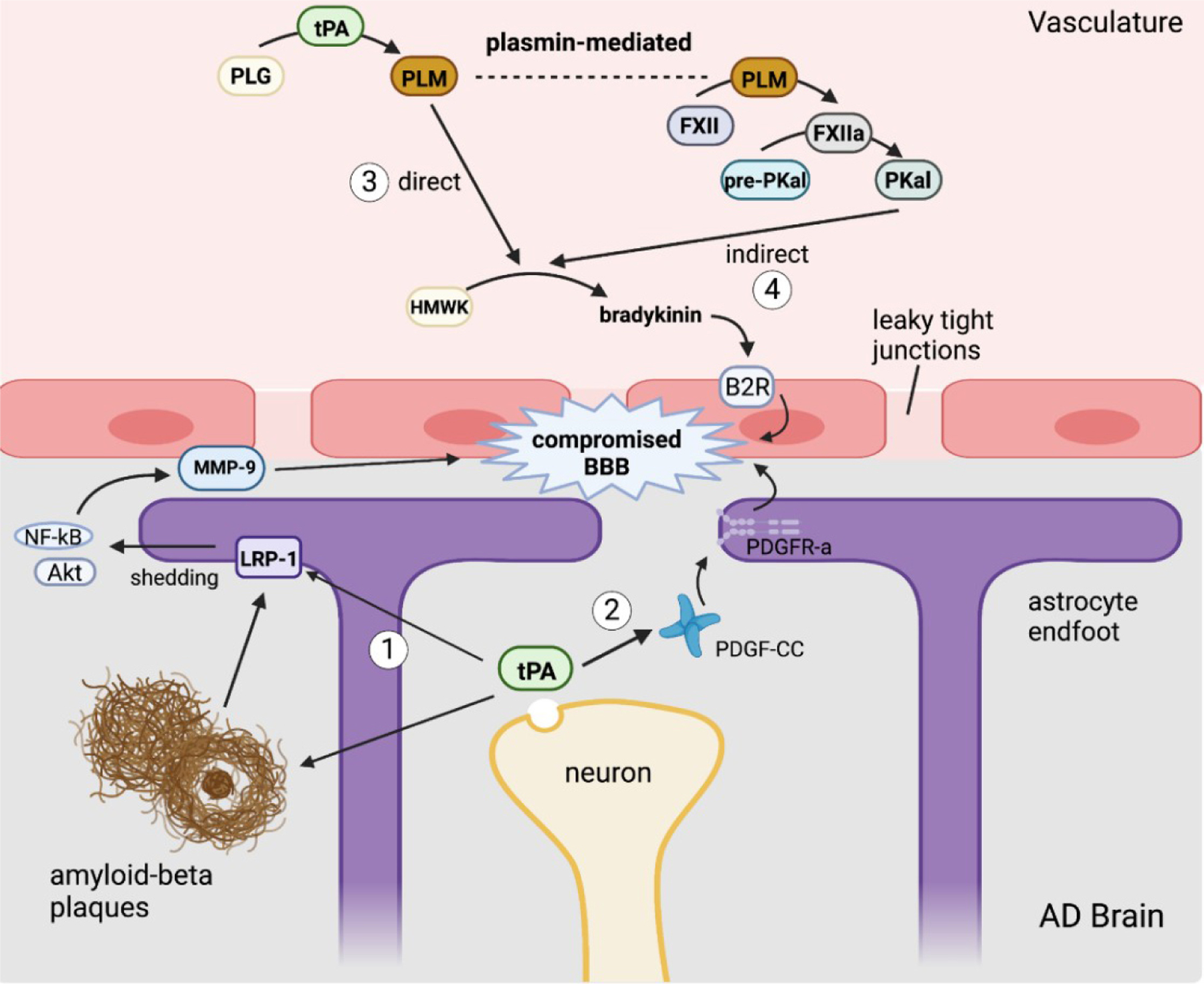
Mechanisms by which tPA may disrupt the blood-brain barrier. (1) tissue-type plasminogen activator(tPA) released from neurons cleaves lipoprotein receptor-related protein-1 (LRP-1) to activate an NF-κB signaling cascade resulting in the production of MMP-9. tPA and LRP-1 can bind amyloid beta, which facilitates Aβ endocytosis across the blood-brain barrier (BBB). (2) Neuronal tPA degrades platelet-derived growth factor-CC (PDGF-CC) to release the active ligand for PDGF receptor-α (PDGFR-α) on astrocytic endfeet, causing them to retract from endothelial cells. (3) Plasma tPA activates plasmin to directly produce bradykinin that activates bradykinin 2 receptor (B2R) receptor on endothelial cells. (4) Plasma tPA cleaves plasminogen to generate plasmin that indirectly upregulates bradykinin expression through plasma kallikrein (PKal). Created with BioRender.com.

In CNS injury, there are several potential mechanisms by which tPA is able to mediate changes in the permeability of the BBB [Figure 3], which in turn further exacerbates CNS injury by promoting neuroinflammation. AD is associated with BBB dysfunction in humans and animal models. Amyloid deposition activates gliosis that can alter the morphology of astrocytic endfeet, which are integral to the integrity of the neurovascular unit. As described previously with CAA, amyloid deposition can also injure the brain endothelium, which can additionally impair BBB integrity^[106]^. Finally, Aβ oligomers stimulate fibrin production that complexes with amyloid plaques, and fibrin has been shown to be increased in the parenchyma and vasculature of AD brains^[107,108]^. This fibrin-Aβ complex promotes further neuroinflammation and neurodegeneration. tPA is conformationally activated by fibrin deposition, but its enzymatic activity is inhibited by the elevated levels of PAI-1 found in AD parenchyma. However, as summarized in Figure 3, activated tPA has multiple plasmin-independent mechanisms by which it can compromise BBB integrity.

#### tPA in the CNS directly alters BBB integrity

tPA has long been known to play a significant role in the NVU, mostly in the context of stroke^[109–111]^. tPA has been reported to directly alter the BBB integrity by triggering activation of LRP-1 on the surface of astrocytes^[12]^. LRP-1 is a multifunctional signaling receptor that functions in receptor-mediated endocytosis and cellular signaling. LRP-1 binds many ligands, including tPA and amyloid-beta^[112]^, which thereby facilitates Aβ endocytosis across endothelial cells of the BBB^[113]^. Aβ oligomers may compromise BBB integrity via activation of matrix metalloproteinases (MMPs)^[113]^. Alternatively, tPA may cleave LRP-1 at its substrate binding ectodomain, activating NF-κB, which promotes the synthesis of matrix metalloproteinases MMP-3 and MMP-9, leading to matrix protein degradation and BBB leakage^[12]^. tPA-induced activation of LRP-1 shedding from astrocytic endfeet also promotes detachment of endfeet projections from tight junctions of the endothelial cells of the neurovascular unit, further compromising the BBB^[12]^. Additionally, tPA can directly alter BBB integrity via platelet-derived growth factor PDGF-CC^[114]^. Upregulated neuronal expression of tPA expression induced by CNS disease or injury results in the release of tPA into the extracellular matrix of the brain, where it cleaves complement subcomponents C1r/C1s, urchin EGF-like protein, and bone-morphogenic protein-1 (CUB) from PDGF-CC forming an active ligand that binds to PDGF receptor-α (PDGFR-α). PDGFR-α promotes BBB leakage that worsens cerebral edema, neuroinflammation and neuronal death^[114]^. One study found this tPA-mediated activation of PDGF-CC to be inefficient in an *in vitro* stroke model^[115]^. However, *in vivo*, the Mac-1 integrin expressed on microglia works cooperatively with the endocytic receptor LRP-1 to promote tPA-mediated activation of PDGF-CC^[115]^. Multiple studies have also implicated tPA in binding amyloid-beta, thereby facilitating Aβ endocytosis across endothelial cells of the BBB^[113]^.

#### Peripheral tPA alters BBB

In addition to its endogenous effects within the CNS, peripheral tPA can cross the intact BBB^[116]^, phosphorylate claudin-5 and occludin, thereby weakening endothelial tight junctions and increasing BBB permeability by plasmin-independent mechanisms^[117,118]^. Chronic release of plasma tPA can induce a hyperfibrinolytic state that also directly increases vascular permeability of the BBB. Resultant plasmin activation by tPA also triggers bradykinin (BK) production^[119,120]^. BK is a peptide mediator generated from its circulating precursor, high molecular weight kininogen (HMWK), and is known for its ability to induce vascular permeability and cause vasodilation of arteries and veins^[119]^. It is a pro-inflammatory mediator, and its role as a neuromediator was identified in clinical conditions including AD^[119]^. While it is still debated as to how the PAS triggers BK generation, two primary pathways have been proposed [Figure 3]. A direct mechanism identified using an *in vitro* model involves tPA-mediated conversion of plasminogen to plasmin, which then cleaves HMWK into BK. BK acts through the bradykinin 2 receptor (B2R) on endothelial cells, triggering a signaling cascade that promotes intracellular calcium release and downregulation of claudin-5, a critical protein involved in maintaining EC tight junctions^[120]^. B2R activation can additionally induce tPA release from endothelial cells, further amplifying additional BK generation^[121]^. The PAS alternatively can indirectly trigger BK formation through a plasmin-dependent pathway where plasmin activated by tPA then converts Factor XII (FXII) into Factor XIIa (FXIIa), which then converts plasma pre-kallikrein into plasma kallikrein (PKal)^[121]^. PKal then serves to cleave HMWK, leading to BK formation and B2R signaling activation [Figure 3]. This indirect mechanism was demonstrated *ex vivo* and *in vivo* with the former using human plasma incubated with tPA, which resulted in the formation of active PKal; the latter demonstrating that intravenous injection of tPA in mice increased PKal activity^[121,122]^.

AD has been shown to produce BBB dysfunction in humans and animal models. Amyloid deposition activates gliosis that can alter the morphology of astrocytic endfeet, which are integral to the integrity of the neurovascular unit. As described previously with CAA, amyloid deposition can injure the brain endothelium, which can additionally impair BBB integrity^[106]^. Finally, Aβ oligomers stimulate fibrin production that complexes with amyloid plaques and has been shown to be increased in the parenchyma and vasculature of AD brains^[107]^. This fibrin-Aβ complex promotes further neuroinflammation and neurodegeneration. tPA is conformationally activated by fibrin deposition, but its enzymatic activity is inhibited by the elevated levels of PAI-1 found in AD parenchyma. However, as summarized in Figure 3, activated tPA has multiple plasmin-independent mechanisms by which it can compromise BBB integrity.

## CONCLUSION

Over the past two decades following initial reports of histologic evidence of Aβ deposition in the brains of children chronically exposed to severe air pollution^[123]^, it has become clear that chronic peripheral inflammatory conditions, including those that involve lung, gut, liver, and metabolic syndrome, exacerbate or initiate neuroinflammatory disorders. This has been supported by epidemiologic findings of a positive association between chronic peripheral inflammatory conditions and increased incidence of dementia, including AD. More recently, there has been increased interest in the contribution of the peripheral PAS to the neuroinflammatory component of AD. Recently, it has become recognized that the risk of blood clots, increased mortality, and persistent neuroinflammatory complications of COVID 19 viral infections are also associated with pre-existing systemic inflammatory disorders shown to chronically activate components of the PAS^[124]^. With respect to AD, the available evidence suggests that the peripheral PAS may modulate the neuroinflammatory response via multiple mechanisms^[12,51]^. Besides fostering the transcytosis of inflammatory cells across the BBB, components of the PAS have been shown to decrease BBB integrity and increase BBB permeability, consequences that have been independently linked to early cognitive dysfunction^[125]^ including progressive stages of AD^[126]^ perhaps in association with concomitant vascular disease^[127]^. Overall, the means by which the PAS modulates BBB integrity by tPA and plasmin-dependent mechanisms is complex and requires further validation and investigation. tPA in the CNS has been shown to alter BBB permeability by LRP-1 and PDGF-CC-dependent mechanisms, while tPA produced from peripheral inflammation can cross the BBB where it may work in tandem with the kinin system to directly generate BK via plasmin, or indirectly by increased PKal. It is likely that tPA works multifactorially and that these mechanisms are not mutually exclusive [Figure 2]^[118]^. Based on what is currently known, further studies investigating the role of the PAS in AD and other dementias are certainly warranted.
